# Supplementary material for: Lack of genetic structure in greylag goose (Anser anser) populations along the European Atlantic flyway
Source: PeerJ. 2015 Aug 13;3:e1161. doi: 10.7717/peerj.1161 (PMC4558074; doi:10.7717/peerj.1161)
Supplement: Appendix S1 [file peerj-03-1161-s001.pdf]

Appendix 1 - List of samples used in this study, with ID, population, sample provenance, collecting date, haplotype assignment and whether the sample was genotyped with microsatellites (STRs).

Sample	ID	POP	pop_name	Country	Locality	Sampling date	MtDNA Haplotype	STRs
aa68	PB/87	NL	Netherlands	Netherlands	Netherlands	13/10/11	ANS15	
aa69	PB/80	NL	Netherlands	Netherlands	Netherlands	13/10/11	ANS5	x
aa70	PB/86	NL	Netherlands	Netherlands	Netherlands	30/09/11	ANS3	x
aa71	PB/75	NL	Netherlands	Netherlands	Netherlands	15/09/11	ANS15	x
aa72	PB/81	NL	Netherlands	Netherlands	Netherlands	30/09/11	ANS3	x
aa73	PB/79	NL	Netherlands	Netherlands	Netherlands	30/09/11	ANS6	x
aa74	PB/85	NL	Netherlands	Netherlands	Netherlands	13/10/11	ANS3	x
aa75	PB/76	NL	Netherlands	Netherlands	Netherlands	27/09/11	ANS1	x
aa76	PB/78	NL	Netherlands	Netherlands	Netherlands	27/09/11	ANS7	x
aa77	PB/82	NL	Netherlands	Netherlands	Netherlands	27/09/11	ANS1	x
aa78	PB/88	NL	Netherlands	Netherlands	Netherlands	27/09/11	ANS3	x
aa79	PB/77	NL	Netherlands	Netherlands	Netherlands	30/09/11	ANS10	x
aa80	PB/83	NL	Netherlands	Netherlands	Netherlands	20/09/11	ANS1	x
aa81	PB/74	NL	Netherlands	Netherlands	Netherlands	15/09/11		x
aa82	PB/84	NL	Netherlands	Netherlands	Netherlands	20/09/11	ANS9	x
aa26	59/33	59/62	Nord	Northern France	Nord	2011/2012	ANS1	x
aa37	59/40	59/62	Nord	Northern France	Nord	2011/2012	ANS13	x
aa41	59/44	59/62	Nord	Northern France	Nord	2011/2012		x
aa43	59/32	59/62	Nord	Northern France	Nord	2011/2012	ANS1	x
aa48	59/31	59/62	Nord	Northern France	Nord			x
aa53	59/45	59/62	Nord	Northern France	Nord		ANS1	
aa58	59/34	59/62	Nord	Northern France	Nord		ANS1	x
aa67	59/30	59/62	Nord	Northern France	Nord	end Jan 2012		x
aa103	59/89	59/62	Nord	Northern France	Nord	15/10/11	ANS4	x
aa104	59/90	59/62	Nord	Northern France	Nord		ANS15	
aa105	59/91	59/62	Nord	Northern France	Nord	15/10/11	ANS15	
aa106	59/92	59/62	Nord	Northern France	Nord		ANS1	x
aa107	59/93	59/62	Nord	Northern France	Nord	12/11/11	ANS3	x
aa108	59/94	59/62	Nord	Northern France	Nord	12/11/11	ANS3	x
aa109	59/95	59/62	Nord	Northern France	Nord	12/11/11	ANS10	x
aa110	59/96	59/62	Nord	Northern France	Nord	12/11/11	ANS3	x
aa111	59/97	59/62	Nord	Northern France	Nord	06/11/11	ANS3	x

aa112	59/98	59/62	Nord	Northern France	Nord	24/10/11	ANS10		x
aa113	59/99	59/62	Nord	Northern France	Nord	24/10/11	ANS10		x
aa49	60/43	60/76/80	Oise	Northern France	Oise		ANS3		x
aa93	60/60	60/76/80	Oise	Northern France	Oise	10/02/12			x
aa94	60/55	60/76/80	Oise	Northern France	Oise	10/02/12	ANS10		x
aa95	60/54	60/76/80	Oise	Northern France	Oise	10/02/12	ANS4		x
aa96	60/59	60/76/80	Oise	Northern France	Oise	04/02/12	ANS12		x
aa97	60/58	60/76/80	Oise	Northern France	Oise	10/02/12			x
aa98	60/56	60/76/80	Oise	Northern France	Oise	10/02/12	ANS4		x
aa114	60/151	60/76/80	Oise	Northern France	Oise		ANS7		x
aa115	60/152	60/76/80	Oise	Northern France	Oise		ANS1		x
aa116	60/153	60/76/80	Oise	Northern France	Oise		ANS18		x
aa117	60/154	60/76/80	Oise	Northern France	Oise	28/11/10	ANS13		x
aa118	60/155	60/76/80	Oise	Northern France	Oise	28/11/10	ANS13		x
aa56	62/35	59/62	Nord	Northern France	Pas-de-Calais				x
aa62	62/36	59/62	Nord	Northern France	Pas-de-Calais		ANS7		x
aa119	FRA124	60/**76**/80	Oise	Northern France	Seine-Maritime		ANS3		x
aa45	80/37	60/76/80	Oise	Northern France	Somme	2011/2012	ANS6		
aa51	80/38	60/76/80	Oise	Northern France	Somme		ANS10		x
aa120	Fin100	Finm	Finnmark	Norway	Finnmark	01/06/10	ANS3		x
aa121	Fin101	Finm	Finnmark	Norway	Finnmark	01/06/10	ANS3		x
aa122	Fin102	Finm	Finnmark	Norway	Finnmark	01/06/10	ANS3		x
aa123	Fin103	Finm	Finnmark	Norway	Finnmark	01/06/10	ANS3		x
aa124	Fin104	Finm	Finnmark	Norway	Finnmark	01/06/10	ANS18		X
aa126	Fin106	Finm	Finnmark	Norway	Finnmark	01/06/10	ANS3		x
aa127	Fin107	Finm	Finnmark	Norway	Finnmark	01/06/10	ANS3		x
aa128	Fin108	Finm	Finnmark	Norway	Finnmark	01/06/10	ANS3		x
aa129	Fin109	Finm	Finnmark	Norway	Finnmark	01/06/10	ANS19		x
aa130	Fin110	Finm	Finnmark	Norway	Finnmark	01/06/10	ANS18		x
aa131	Fin111	Finm	Finnmark	Norway	Finnmark	01/06/10	ANS18		x
aa132	Veg113	Norw	Vega	Norway	Vega	01/06/10	ANS3		x
aa133	Veg116	Norw	Vega	Norway	Vega	01/06/10	ANS3		x
aa134	Veg123	Norw	Vega	Norway	Vega	01/06/10	ANS3		x
aa135	Veg128	Norw	Vega	Norway	Vega	01/06/10	ANS3		x
aa136	Veg131	Norw	Vega	Norway	Vega	01/06/10	ANS3		x
aa137	Veg132	Norw	Vega	Norway	Vega	01/06/10	ANS3		x
aa138	Veg134	Norw	Vega	Norway	Vega	01/06/10	ANS3		x
aa139	Veg135	Norw	Vega	Norway	Vega	01/06/10			x

aa140	Veg137	Norw	Vega	Norway	Vega	01/06/10		x
aa141	Veg140	Norw	Vega	Norway	Vega	01/06/10	ANS3	x
aa142	Veg141	Norw	Vega	Norway	Vega	01/06/10	ANS3	x
aa143	Smo112	Norw	Vega	Norway	Vega	01/06/10		x
aa144	Smo114	Norw	Vega	Norway	Vega	01/06/10		x
aa145	Smo115	Norw	Vega	Norway	Vega	01/06/10		x
aa146	Smo117	Norw	Vega	Norway	Vega	01/06/10	ANS3	x
aa147	Smo118	Norw	Vega	Norway	Vega	01/06/10	ANS3	x
aa148	Smo119	Norw	Vega	Norway	Vega	01/06/10		x
aa149	Smo120	Norw	Vega	Norway	Vega	01/06/10	ANS3	x
aa150	Smo121	Norw	Vega	Norway	Vega	01/06/10		x
aa151	Smo122	Norw	Vega	Norway	Vega	01/06/10	ANS3	x
aa152	Smo125	Norw	Vega	Norway	Vega	01/06/10		x
aa153	Smo126	Norw	Vega	Norway	Vega	01/06/10		x
aa154	Smo127	Norw	Vega	Norway	Vega	01/06/10	ANS3	x
aa155	Smo129	Norw	Vega	Norway	Vega	01/06/10		x
aa156	Smo130	Norw	Vega	Norway	Vega	01/06/10	ANS3	x
aa157	Smo133	Norw	Vega	Norway	Vega	01/06/10	ANS3	x
aa158	Smo136	Norw	Vega	Norway	Vega	01/06/10	ANS3	x
aa159	Smo138	Norw	Vega	Norway	Vega	01/06/10	ANS3	x
aa160	Smo139	Norw	Vega	Norway	Vega	01/06/10	ANS3	x
aa161	Smo142	Norw	Vega	Norway	Vega	01/06/10		x
aa162	Smo146	Norw	Vega	Norway	Vega	01/06/10	ANS15	x
aa163	Smo147	Norw	Vega	Norway	Vega	01/06/10	ANS3	x
aa164	No143	Norw	Vega	Norway	Vega	01/06/10	ANS3	x
aa165	No149	Norw	Vega	Norway	Vega	01/06/10	ANS16	x
aa166	17/160	17	Charente Maritime	South-western France	Charente Maritime	10/02/12	ANS3	x
aa167	17/161	17	Charente Maritime	South-western France	Charente Maritime	07/02/12	ANS7	x
aa168	17/162	17	Charente Maritime	South-western France	Charente Maritime	10/02/12	ANS15	x
aa169	17/163	17	Charente Maritime	South-western France	Charente Maritime	07/02/12	ANS15	x
aa170	17/164	17	Charente Maritime	South-western France	Charente Maritime	07/02/12	ANS20	
aa171	17/165	17	Charente Maritime	South-western France	Charente Maritime	07/02/12	ANS21	x
aa172	17/166	17	Charente Maritime	South-western France	Charente Maritime	07/02/12	ANS21	x
aa173	17/167	17	Charente Maritime	South-western France	Charente Maritime	10/02/12	ANS1	x
aa174	17/168	17	Charente Maritime	South-western France	Charente Maritime	10/02/12	ANS22	x
aa175	17/169	17	Charente Maritime	South-western France	Charente Maritime	05/02/12	ANS3	x
aa16	33/09	33	Gironde	South-western France	Gironde	end Jan 2012	ANS4	x
aa17	33/05	33	Gironde	South-western France	Gironde	end Jan 2012		x

aa18	33/11	33	Gironde	South-western France	Gironde	end Jan 2012	ANS1	x
aa19	33/12	33	Gironde	South-western France	Gironde	end Jan 2012	ANS1	x
aa22	33/25	33	Gironde	South-western France	Gironde	end Jan 2012	ANS1	x
aa23	33/03	33	Gironde	South-western France	Gironde	end Jan 2012		x
aa25	33/18	33	Gironde	South-western France	Gironde	08/11/11	ANS15	
aa27	33/08	33	Gironde	South-western France	Gironde	end Jan 2012	ANS3	x
aa28	33/02	33	Gironde	South-western France	Gironde	end Jan 2012	ANS7	x
aa29	33/07	33	Gironde	South-western France	Gironde	end Jan 2012	ANS16	x
aa30	33/17	33	Gironde	South-western France	Gironde	end Jan 2012		x
aa31	33/04	33	Gironde	South-western France	Gironde	end Jan 2012	ANS1	x
aa32	33/19	33	Gironde	South-western France	Gironde	end Jan 2012	ANS1	x
aa33	33/06	33	Gironde	South-western France	Gironde	end Jan 2012	ANS1	x
aa36	33/14	33	Gironde	South-western France	Gironde	end Jan 2012	ANS3	x
aa38	33/13	33	Gironde	South-western France	Gironde	end Jan 2012	ANS7	x
aa39	33/20	33	Gironde	South-western France	Gironde	end Jan 2012	ANS17	x
aa40	33/26	33	Gironde	South-western France	Gironde	end Jan 2012	ANS15	x
aa42	33/27	33	Gironde	South-western France	Gironde	end Jan 2012	ANS1	x
aa44	33/21	33	Gironde	South-western France	Gironde	end Jan 2012	ANS3	x
aa59	33/29	33	Gironde	South-western France	Gironde	end Jan 2012		x
aa60	33/23	33	Gironde	South-western France	Gironde	end Jan 2012	ANS15	x
aa64	33/24	33	Gironde	South-western France	Gironde	end Jan 2012	ANS3	x
aa65	33/16	33	Gironde	South-western France	Gironde	end Jan 2012		x
aa66	33/22	33	Gironde	South-western France	Gironde	end Jan 2012	ANS15	x
aa24	40/47	40/64	Landes	South-western France	Landes	08/11/11	ANS15	x
aa34	X/53	40/64	Landes	South-western France	Landes	2011/2012	ANS17	x
aa35	X/50	40/64	Landes	South-western France	Landes	2011/2012	ANS1	x
aa46	40/46	40/64	Landes	South-western France	Landes	14/12/11	ANS3	x
aa52	X/39	40/64	Landes	South-western France	Landes	_		x
aa54	X/49	40/64	Landes	South-western France	Landes	_	ANS13	x
aa55	40/42	40/64	Landes	South-western France	Landes	_	ANS15	x
aa61	X/41	40/64	Landes	South-western France	Landes	_	ANS15	x
aa83	40/73	40/64	Landes	South-western France	Landes	16/11/11	ANS1	x
aa84	40/69	40/64	Landes	South-western France	Landes	16/11/11	ANS3	x
aa85	40/61	40/64	Landes	South-western France	Landes	29/01/12	ANS11	x
aa86	40/65	40/64	Landes	South-western France	Landes	16/11/11		x
aa87	40/71	40/64	Landes	South-western France	Landes	16/11/11		x
aa89	40/70	40/64	Landes	South-western France	Landes	16/11/11	ANS1	x
aa90	40/72	40/64	Landes	South-western France	Landes	16/11/11	ANS15	

aa91	40/67	40/64	Landes	South-western France	Landes	04/02/12		x
aa100	X/66	40/64	Landes	South-western France	Landes	_		x
aa101	X/62	40/64	Landes	South-western France	Landes	_		x
aa102	X/63	40/64	Landes	South-western France	Landes	_		x
aa176	40/170	40/64	Landes	South-western France	Landes	02/02/12	ANS21	x
aa178	40/172	40/64	Landes	South-western France	Landes	09/02/12	ANS1	x
aa180	40/174	40/64	Landes	South-western France	Landes	04/02/12	ANS23	x
aa181	40/175	40/64	Landes	South-western France	Landes	09/02/12	ANS15	x
aa182	40/176	40/64	Landes	South-western France	Landes	02/02/12	ANS1	x
aa183	40/177	40/64	Landes	South-western France	Landes	01/02/12	ANS3	x
aa184	40/178	40/64	Landes	South-western France	Landes	01/02/12	ANS1	x
aa185	40/179	40/64	Landes	South-western France	Landes	03/02/12	ANS1	x
aa186	40/180	40/64	Landes	South-western France	Landes	03/02/12	ANS14	x
aa187	40/181	40/64	Landes	South-western France	Landes	10/02/12	ANS20	x
aa188	40/182	40/64	Landes	South-western France	Landes	10/02/12	ANS7	x
aa189	40/183	40/64	Landes	South-western France	Landes	12/02/12	ANS14	x
aa190	40/184	40/64	Landes	South-western France	Landes	12/02/12	ANS1	x
aa40-1		40/64	Landes	South-western France	Landes	_	ANS1	x
aa40-2		40/64	Landes	South-western France	Landes	_	ANS1	x
aa40-3		40/64	Landes	South-western France	Landes	_	ANS1	x
aa40-4		40/64	Landes	South-western France	Landes	_	ANS2	x
aa40-5		40/64	Landes	South-western France	Landes	_	ANS8	x
aa40-6		40/64	Landes	South-western France	Landes	_	ANS1	X
aa40-7		40/64	Landes	South-western France	Landes	_	ANS1	X
aa40-8		40/64	Landes	South-western France	Landes	_	ANS1	X
aa47	64/52	40/64	Landes	South-western France	Pyrenee Atlantique	_	ANS15	x
aa50	64/48	40/64	Landes	South-western France	Pyrenee Atlantique	_	ANS1	x
aa57	64/51	40/64	Landes	South-western France	Pyrenee Atlantique	_	ANS3	x
rub1				Russia	Kalmykia		17	
