# Supplementary material for: Lack of genetic structure in greylag goose (Anser anser) populations along the European Atlantic flyway
Source: PeerJ. 2015 Aug 13;3:e1161. doi: 10.7717/peerj.1161 (PMC4558074; doi:10.7717/peerj.1161)
Supplement: Appendix S2 [file peerj-03-1161-s002.pdf]

	France					North Europe			
	**Charente Maritime**	**Gironde**	**Landes**	**Nord**	**Oise**	**Norway Finnmark**	**Norway Vega**	**Netherlands**	**Total**
**ANS1**	1	7	15	5	1			3	32
**ANS2**			1						1
**ANS3**	2	4	4	4	2	7	21	4	48
**ANS4**		1		1	2				4
**ANS5**								1	1
**ANS6**					1			1	2
**ANS7**	1	2	1	1	1			1	7
**ANS8**			1						1
**ANS9**								1	1
**ANS10**				3	2			1	6
**ANS11**			1						1
**ANS12**					1				1
**ANS13**			1	1	2				4
**ANS14**			2						2
**ANS15**	2	4	6	2			1	2	17
**ANS16**		1					1		2
**ANS17**		1	1						2
**ANS18**					1	3			4
**ANS19**						1			1
**ANS20**	1		1						2
**ANS21**	2		1						3
**ANS22**	1								1
**ANS23**			1						1
Total	10	20	36	17	13	11	23	14	144
