# Supplementary material for: Lack of genetic structure in greylag goose (Anser anser) populations along the European Atlantic flyway
Source: PeerJ. 2015 Aug 13;3:e1161. doi: 10.7717/peerj.1161 (PMC4558074; doi:10.7717/peerj.1161)
Supplement: Appendix S3 [file peerj-03-1161-s003.pdf]

Appendix 3 – Summary of genetic variation at 14 microsatellite loci in sampled populations. Na = No. of different alleles; Ne = No. of effective alleles. Ho = observed heterozygosity; He = expected heterozygosity; F = fixation index; HWE = Hardy-Weinberg equilibrium.

Pop	Locus	N	Na	Ne	Ho	He	F
**Netherlands**	**Ans02**	13	9.000	4.390	0.692	0.772	0.103
	**Ans04**	14	3.000	2.947	0.214	0.661	0.676
	**Ans07**	14	3.000	1.782	0.286	0.439	0.349
	**Ans17**	14	7.000	4.667	0.643	0.786	0.182
	**Ans18**	14	4.000	2.190	0.214	0.543	0.606
	**Ans21**	14	3.000	1.640	0.500	0.390	-0.281
	**Ans24**	14	3.000	1.338	0.286	0.253	-0.131
	**Ans25**	14	6.000	3.769	0.786	0.735	-0.069
	**Aalu1b**	14	4.000	2.190	0.429	0.543	0.211
	**Aph12**	14	2.000	1.600	0.214	0.375	0.429
	**Aph19**	14	2.000	1.774	0.500	0.436	-0.146
	**Smo7**	14	2.000	1.508	0.286	0.337	0.152
	**Hhiu1b**	14	3.000	1.156	0.929	0.135	-5.868
	**Ans13**	14	4.000	1.980	0.286	0.495	0.423
**Nord**	**Ans02**	16	8.000	3.606	0.750	0.723	-0.038
	**Ans04**	17	4.000	2.934	0.706	0.659	-0.071
	**Ans07**	17	3.000	2.181	0.294	0.542	0.457
	**Ans17**	17	8.000	5.026	0.706	0.801	0.119
	**Ans18**	16	3.000	1.724	0.125	0.420	0.702
	**Ans21**	17	3.000	1.847	0.235	0.458	0.487
	**Ans24**	17	1.000	1.000	0.000	0.000	-
	**Ans25**	17	7.000	4.983	0.882	0.799	-0.104
	**Aalu1b**	17	4.000	3.778	0.824	0.735	-0.120
	**Aph12**	15	3.000	2.103	0.533	0.524	-0.017
	**Aph19**	17	2.000	1.973	0.412	0.493	0.165
	**Smo7**	17	2.000	1.710	0.412	0.415	0.008
	**Hhiu1b**	17	4.000	1.889	0.529	0.471	-0.125
	**Ans13**	17	4.000	2.766	0.353	0.638	0.447
**Oise**	**Ans02**	14	9.000	6.759	0.857	0.852	-0.006
	**Ans04**	15	4.000	3.147	0.267	0.682	0.609
	**Ans07**	14	2.000	1.912	0.214	0.477	0.551
	**Ans17**	15	6.000	4.206	0.467	0.762	0.388
	**Ans18**	14	6.000	2.667	0.357	0.625	0.429
	**Ans21**	15	3.000	1.779	0.200	0.438	0.543
	**Ans24**	15	2.000	1.069	0.067	0.064	-0.034
	**Ans25**	15	6.000	3.947	0.600	0.747	0.196
	**Aalu1b**	14	4.000	2.947	0.643	0.661	0.027
	**Aph12**	14	3.000	2.074	0.286	0.518	0.448
	**Aph19**	15	2.000	1.867	0.333	0.464	0.282
	**Smo7**	15	2.000	1.642	0.533	0.391	-0.364
	**Hhiu1b**	15	2.000	1.069	0.067	0.064	-0.034
	**Ans13**	14	3.000	2.741	0.500	0.635	0.213

**Finnmark**	**Ans02**	9	6.000	3.857	0.778	0.741	-0.050
	**Ans04**	10	4.000	2.597	0.400	0.615	0.350
	**Ans07**	10	3.000	2.151	0.200	0.535	0.626
	**Ans17**	11	5.000	3.270	0.818	0.694	-0.179
	**Ans18**	8	2.000	1.280	0.250	0.219	-0.143
	**Ans21**	11	3.000	2.283	0.364	0.562	0.353
	**Ans24**	10	1.000	1.000	0.000	0.000	-
	**Ans25**	10	4.000	2.667	0.700	0.625	-0.120
	**Aalu1b**	11	4.000	1.330	0.182	0.248	0.267
	**Aph12**	11	3.000	1.582	0.455	0.368	-0.236
	**Aph19**	11	3.000	1.603	0.273	0.376	0.275
	**Smo7**	11	2.000	1.198	0.000	0.165	1.000
	**Hhiu1b**	11	2.000	1.198	0.182	0.165	-0.100
	**Ans13**	11	5.000	3.903	0.636	0.744	0.144
**Vega**	**Ans02**	31	11.000	3.979	0.774	0.749	-0.034
	**Ans04**	34	4.000	3.129	0.382	0.680	0.438
	**Ans07**	31	5.000	2.023	0.355	0.506	0.298
	**Ans17**	34	5.000	2.919	0.588	0.657	0.105
	**Ans18**	33	6.000	2.209	0.273	0.547	0.502
	**Ans21**	31	3.000	1.795	0.613	0.443	-0.384
	**Ans24**	30	3.000	1.224	0.067	0.183	0.635
	**Ans25**	34	8.000	5.639	0.882	0.823	-0.073
	**Aalu1b**	34	4.000	2.305	0.588	0.566	-0.039
	**Aph12**	34	3.000	2.070	0.412	0.517	0.203
	**Aph19**	34	2.000	1.895	0.588	0.472	-0.245
	**Smo7**	34	2.000	1.524	0.206	0.344	0.401
	**Hhiu1b**	34	3.000	1.433	0.353	0.302	-0.167
	**Ans13**	34	5.000	2.645	0.588	0.622	0.054
**Charente-Maritime**	**Ans02**	9	8.000	5.400	0.889	0.815	-0.091
	**Ans04**	9	4.000	2.746	0.111	0.636	0.825
	**Ans07**	9	3.000	1.588	0.000	0.370	1.000
	**Ans17**	9	5.000	3.057	0.444	0.673	0.339
	**Ans18**	9	3.000	2.793	0.222	0.642	0.654
	**Ans21**	9	3.000	1.976	0.667	0.494	-0.350
	**Ans24**	9	2.000	1.385	0.333	0.278	-0.200
	**Ans25**	8	6.000	4.923	0.875	0.797	-0.098
	**Aalu1b**	9	4.000	2.282	0.556	0.562	0.011
	**Aph12**	9	2.000	1.800	0.778	0.444	-0.750
	**Aph19**	9	2.000	1.800	0.222	0.444	0.500
	**Smo7**	9	2.000	1.385	0.889	0.278	-2.200
	**Hhiu1b**	9	3.000	1.256	0.222	0.204	-0.091
	**Ans13**	9	3.000	2.160	0.556	0.537	-0.034
**Gironde**	**Ans02**	23	9.000	5.186	0.826	0.807	-0.023
	**Ans04**	24	4.000	3.892	0.333	0.743	0.551
	**Ans07**	24	4.000	1.466	0.208	0.318	0.344
	**Ans17**	24	7.000	3.959	0.542	0.747	0.275
	**Ans18**	23	5.000	1.853	0.217	0.460	0.528
	**Ans21**	23	3.000	1.772	0.348	0.436	0.202
	**Ans24**	23	1.000	1.000	0.000	0.000	-

	**Ans25**	24	8.000	4.645	0.750	0.785	0.044	
	**Aalu1b**	24	5.000	2.743	0.667	0.635	-0.049	
	**Aph12**	24	2.000	1.946	0.667	0.486	-0.371	
	**Aph19**	23	2.000	1.830	0.435	0.454	0.042	
	**Smo7**	24	2.000	1.180	0.583	0.153	-2.818	
	**Hhiu1b**	24	4.000	1.550	0.333	0.355	0.061	
	**Ans13**	23	4.000	3.032	0.565	0.670	0.157	
**Landes**	**Ans02**	45	11.000	4.500	0.756	0.778	0.029	
	**Ans04**	44	4.000	3.741	0.477	0.733	0.349	
	**Ans07**	44	3.000	1.955	0.227	0.488	0.535	
	**Ans17**	45	7.000	4.787	0.689	0.791	0.129	
	**Ans18**	45	6.000	1.777	0.333	0.437	0.238	
	**Ans21**	45	3.000	1.874	0.467	0.466	-0.001	
	**Ans24**	45	3.000	1.280	0.222	0.219	-0.015	
	**Ans25**	45	8.000	4.350	0.800	0.770	-0.039	
	**Aalu1b**	45	5.000	3.061	0.489	0.673	0.274	
	**Aph12**	45	2.000	1.776	0.378	0.437	0.135	
	**Aph19**	44	3.000	1.890	0.477	0.471	-0.014	
	**Smo7**	45	2.000	1.557	0.556	0.358	-0.553	
	**Hhiu1b**	45	4.000	1.529	0.356	0.346	-0.028	
	**Ans13**	45	4.000	2.675	0.556	0.626	0.113	

		**N**	**Na**	**Ne**	**Ho**	**He**	**F**	**HWE P-value**	**HWE (se)**
**Mean for each locus (all populations confounded)**	**Ans02**	20.000	8.875	4.710	0.790	0.780	-0,014	0.3783	(0.0209)
	**Ans04**	20.875	3.875	3.142	0.361	0.676	0,466	0.0000	(0.0000)
	**Ans07**	20.375	3.250	1.882	0.223	0.459	0,520	0.0000	(0.0000)
	**Ans17**	21.125	6.250	3.986	0.612	0.739	0,170	0.0000	(0.0000)
	**Ans18**	20.250	4.375	2.062	0.249	0.487	0,439	0.0000	(0.0000)
	**Ans21**	20.625	3.000	1.871	0.424	0.461	0,071	0.1042	(0.003)
	**Ans24**	20.375	2.000	1.162	0.122	0.125	0,051	0.0558	(0.0022)
	**Ans25**	20.875	6.625	4.365	0.784	0.760	-0,033	0.3506	(0.0153)
	**Aalu1b**	21.000	4.250	2.579	0.547	0.578	0,073	0.0218	(0.0022)
	**Aph12**	20.750	2.500	1.869	0.465	0.459	-0,020	0.0406	(0.0015)
	**Aph19**	20.875	2.250	1.829	0.405	0.451	0,107	0.1316	(0.0038)
	**Smo7**	21.125	2.000	1.463	0.433	0.305	-0,547	0.0003	(0.0001)
	**Hhiu1b**	21.125	3.125	1.385	0.371	0.255	-0,794	0.0707	(0.0034)
	**Ans13**	20.875	4.000	2.738	0.505	0.621	0,190	0.0004	(0.0002)
