# Supplementary material for: Lack of genetic structure in greylag goose (Anser anser) populations along the European Atlantic flyway
Source: PeerJ. 2015 Aug 13;3:e1161. doi: 10.7717/peerj.1161 (PMC4558074; doi:10.7717/peerj.1161)
Supplement: Appendix S4 [file peerj-03-1161-s004.pdf]

Appendix 4 - Mismatch distribution calculated for all individuals and each population.

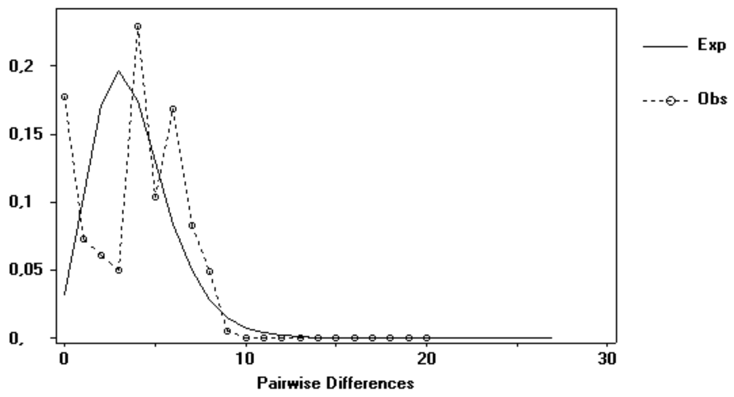

All Samples

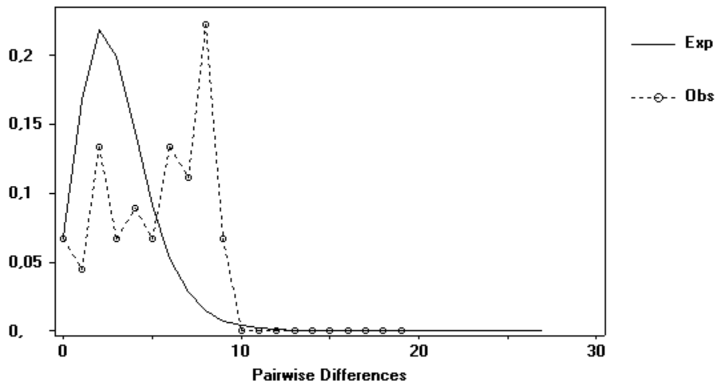

Charente Maritime

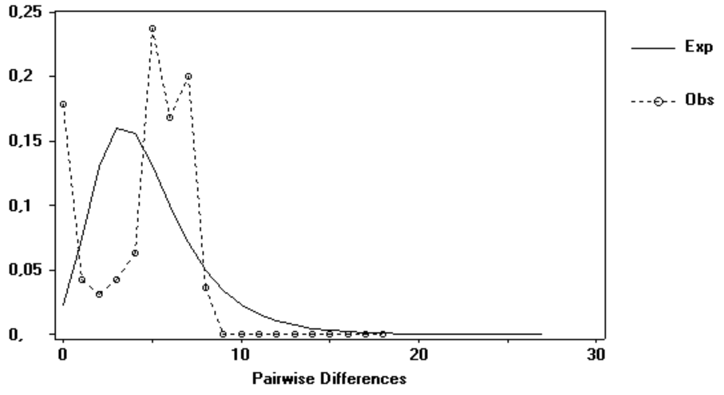

Gironde

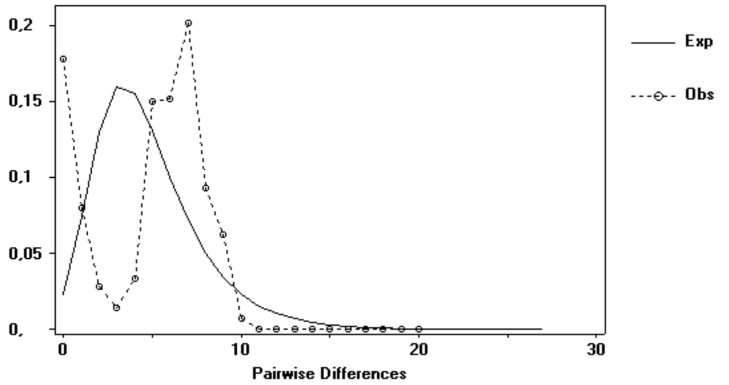

Landes

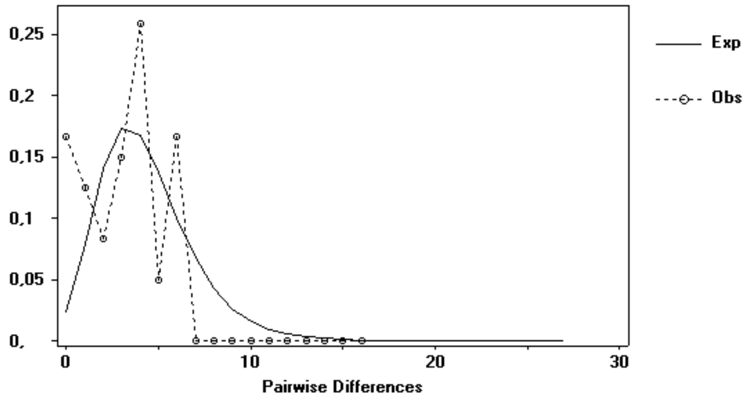

Nord

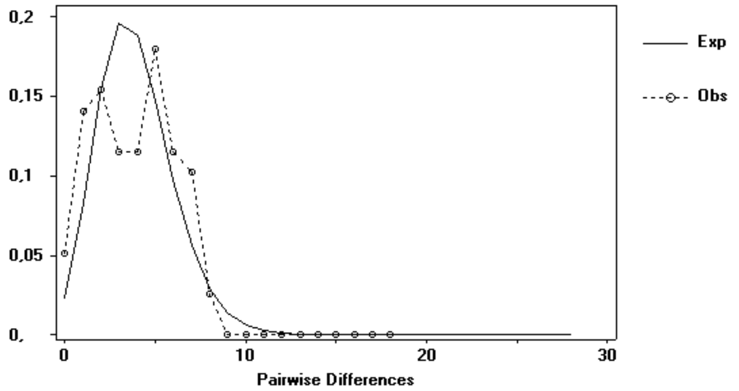

Oise

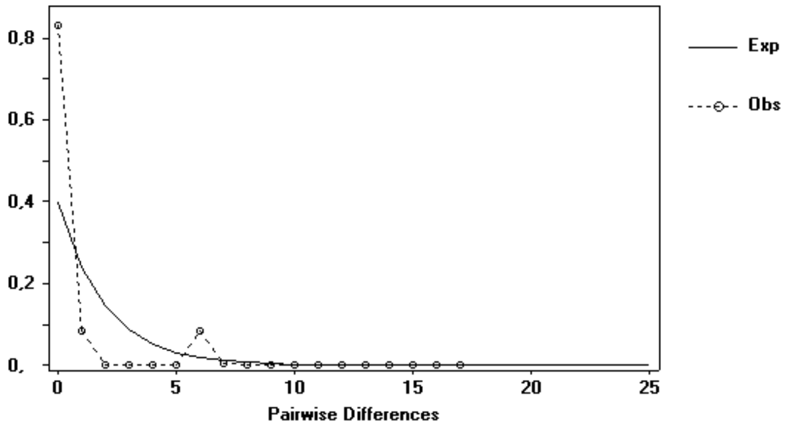

Norway Vega

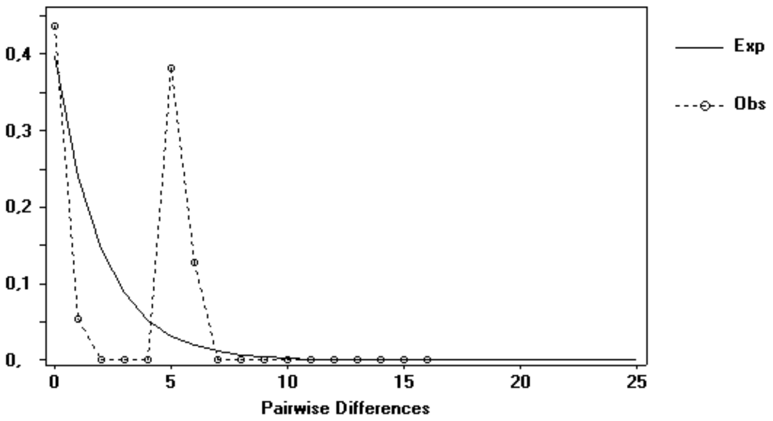

Norway Finnmark

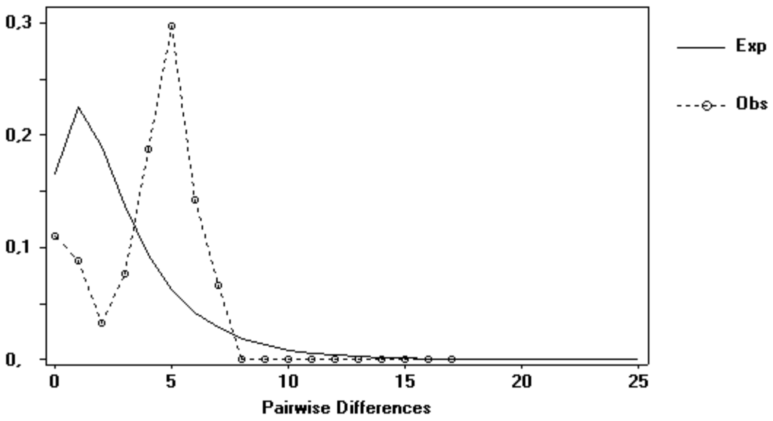

Netherlands
